# Scapular endurance, dyskinesis, and functional outcomes after rotator cuff repair: A cross-sectional mediation analysis

**DOI:** 10.1371/journal.pone.0357765

**Published:** 2026-09-15

**Authors:** Wael M. Alzahrani, Baraa Abdulraheem Baltow, Ziad Ahmed Alanazi, Saleh M. Kardm, Ravi Shankar Reddy, Ghada Mohamed. Koura, Ajay Prashad Gautam

**Affiliations:** 1 Department of Surgery, College of Medicine, Najran University, Najran, Saudi Arabia; 2 Department of Orthopedic Surgery, AlHada Armed Forces Hospital, Taif, Saudi Arabia; 3 Department of Surgery, Orthopedic Division, College of Medicine, Jouf University, Sakaka, Saudi Arabia; 4 Program of Physical Therapy, Department of Medical Rehabilitation Sciences, College of Applied Medical Sciences, King Khalid University, Abha, Saudi Arabia; Brunel University London, UNITED KINGDOM OF GREAT BRITAIN AND NORTHERN IRELAND

## Abstract

This cross-sectional study investigates the relationship between scapular muscle endurance and shoulder function in patients who had undergone arthroscopic rotator cuff repair (RCR) 6–18 months before study enrollment. A total of 110 patients were assessed using the Scapular Retraction Endurance Test (SRET), the Scapular Dyskinesis Test, and the Closed Kinetic Chain Upper Extremity Stability Test (CKCUEST). Patient-reported outcomes were measured using the American Shoulder and Elbow Surgeons (ASES) score, the Disabilities of the Arm, Shoulder and Hand (DASH) questionnaire, and a Numerical Rating Scale (NRS) for pain. Findings revealed that higher scapular endurance and better CKCUEST performance were significantly associated with improved shoulder function (higher ASES scores) and reduced disability (lower DASH scores). Scapular dyskinesis was associated with poorer clinical and functional outcomes and mediated the relationship between scapular endurance and functional status. Mediation analyses confirmed that both scapular dyskinesis and CKCUEST performance partially explained the impact of endurance on functional recovery. The results suggest that scapular muscle endurance is a key predictor of postoperative shoulder function and is influenced by scapular motion quality and upper extremity stability. These findings support the incorporation of endurance training and scapular control assessments into rehabilitation protocols for patients following RCR.

## Background

Rotator cuff tears are among the most prevalent causes of shoulder dysfunction in adults, contributing to pain, limited range of motion, and reduced quality of life [1]. Arthroscopic rotator cuff repair (RCR) is widely regarded as the standard surgical intervention for patients with persistent symptoms and functional deficits unresponsive to conservative management [2]. While surgical repair often restores tendon integrity, a considerable proportion of patients continue to report ongoing pain and functional limitations months after surgery [3]. This disparity between structural healing and clinical recovery has redirected attention toward modifiable neuromuscular and biomechanical factors that influence long-term outcomes [4]. Among these, scapular muscle performance and movement control have consistently been associated with postoperative shoulder function [4]. Given the scapula’s central role in coordinating shoulder mechanics, endurance deficits in scapular stabilizers may compromise rehabilitation progress and functional return [5].

Recent research has highlighted the association between scapular dyskinesis—defined as abnormal scapular motion during arm movement—and impaired shoulder function in both athletic and post-surgical populations [6–8]. Studies by Longo et al. [9] and Teixeira et al. [8] have shown that scapular dyskinesis can persist after RCR and is frequently associated with altered kinematics, reduced muscle activation, and impaired force coupling. Functional tests such as the Closed Kinetic Chain Upper Extremity Stability Test (CKCUEST) and observational dyskinesis screening have been adopted in clinical settings to assess these impairments [10,11]. Additionally, scapular endurance, typically assessed through sustained retraction tasks, has been recognized as crucial for dynamic shoulder control, particularly during repetitive or overhead activities [11]. Timmons et al. (2012) demonstrated that scapular muscle fatigue significantly disrupts normal scapulohumeral rhythm, which may further contribute to subjective dysfunction [12]. Despite these insights, most studies have focused on scapular strength or positional analysis, with limited integration of endurance capacity into predictive models of patient-reported outcomes [13]. Moreover, the functional implications of scapular kinematic indicators, such as dyskinesis and CKCUEST performance, remain underexplored within multivariate and mechanistic frameworks.

Although scapular strength and control have received growing attention in rehabilitation research, the specific contribution of scapular muscle endurance to postoperative shoulder function has not been sufficiently quantified [14]. Furthermore, while individual links between scapular endurance, movement quality, and function have been reported, few studies have concurrently modeled their interrelationships using standard outcome measures such as the American Shoulder and Elbow Surgeons (ASES) score or the Disabilities of the Arm, Shoulder, and Hand (DASH) questionnaire [14,15]. Understanding whether scapular dyskinesis and upper extremity performance mediate the association between scapular endurance and clinical outcomes is particularly relevant for optimizing rehabilitation strategies in the months following RCR [15]. Given the persistent functional variability observed in this population, there is a compelling need for research that integrates objective performance measures, observational kinematics, and patient-reported disability within a unified clinical framework [16].

The primary objective of this study was to evaluate the association between scapular muscle endurance and shoulder-specific functional outcomes among patients 6–18 months following primary arthroscopic rotator cuff repair. A secondary objective was to examine whether scapular dyskinesis and closed-chain upper extremity performance mediate this relationship. It was hypothesized that (i) greater scapular endurance would be associated with higher ASES scores, lower DASH scores, and reduced pain intensity, and that (ii) scapular dyskinesis and CKCUEST performance would demonstrate indirect effects statistically consistent with partial mediation of these associations; however, these analyses were considered exploratory because the cross-sectional design does not permit causal inference.

## Materials and methods

### Study design, ethics, and settings

This cross-sectional clinical performance study was conducted from 5^th^ May 2024–10^th^ April 2025, at the Orthopedic Rehabilitation Clinic, Department of Physical Therapy and Health Rehabilitation Sciences, King Khalid University, Abha, Kingdom of Saudi Arabia. The design incorporated both observational and analytical components, using multivariable regression and mediation models to evaluate associations and indirect effects. Ethical approval was obtained from the Institutional Review Board of King Khalid University (KKU-165-2025-31), and written informed consent was obtained from all participants before enrollment. The study adhered strictly to the ethical standards outlined in the Declaration of Helsinki.

### Sample size calculation

The sample size was calculated using G*Power based on a moderate effect size (f² = 0.15), five predictors (scapular endurance, scapular dyskinesis, CKCUEST performance, age, and sex), an alpha of 0.05, and a power of 0.90. The analysis indicated that 103 participants were needed, and to account for potential data loss, the final target was set at 110 participants.

### Participants

Participants were recruited through consecutive sampling from the outpatient shoulder rehabilitation service at the Orthopedic Rehabilitation Clinic. Eligible individuals were identified from post-surgical follow-up lists and referred by orthopedic surgeons overseeing postoperative care. All participants had undergone unilateral arthroscopic rotator cuff repair (RCR) performed by board-certified orthopedic surgeons, with surgery completed between 6 and 18 months before enrollment. The diagnosis of a rotator cuff tear was confirmed preoperatively by a combination of clinical examination and imaging, including magnetic resonance imaging (MRI), in accordance with the American Academy of Orthopaedic Surgeons’ diagnostic criteria [17,18].

Inclusion criteria comprised adults aged 40–75 years with a primary diagnosis of full-thickness supraspinatus or infraspinatus tear treated by arthroscopic RCR, currently engaged in postoperative rehabilitation, or discharged within the previous six months. Participants were required to have no ongoing neurological or systemic conditions affecting the upper extremity and sufficient cognitive ability to follow instructions and complete patient-reported outcome measures. Individuals were excluded if they had undergone bilateral shoulder surgery, revision RCR, concurrent labral repair or biceps tenodesis, or presented with adhesive capsulitis, glenohumeral arthritis, or any neuromuscular disorders affecting scapular control.

All eligible participants underwent a screening interview and a chart review to confirm their surgical history and ensure inclusion criteria were met. Baseline assessments, including the collection of demographic and clinical data, were completed before the performance-based and questionnaire-based outcome measures to ensure standardized and unbiased data acquisition.

### Shoulder function and disability

The primary outcomes of interest were patient-reported shoulder function and disability, assessed using the American Shoulder and Elbow Surgeons (ASES**) Standardized Shoulder Assessment Form [19]** and the Disabilities of the Arm, Shoulder and Hand (DASH) **questionnaire** [20]. The ASES score is a composite tool combining patient-reported pain (50 points) and functional ability (50 points), yielding a total score ranging from 0 to 100, with higher scores indicating better shoulder function [19]. The DASH score is a validated 30-item questionnaire that quantifies upper-extremity disability and symptoms across daily tasks, with scores ranging from 0 (no disability) to 100 (maximum disability) [20]. Both instruments have demonstrated excellent reliability and responsiveness in postoperative rotator cuff populations and were administered in their validated Arabic versions under standardized supervision.

### Pain intensity

Pain intensity was measured using the Numerical Rating Scale (NRS) [21], a unidimensional scale ranging from 0 (no pain) to 10 (worst imaginable pain). Participants were asked to rate their average shoulder pain over the past 24 hours during routine activity [21]. The NRS was chosen for its ease of administration and established validity in musculoskeletal and postoperative populations, including those with shoulder conditions.

### Scapular muscle endurance

Scapular muscle endurance was assessed using the Scapular Retraction Endurance Test (SRET), a validated clinical tool that quantifies the endurance capacity of the scapular stabilizers, particularly the middle trapezius and rhomboid muscles [22]. The test was conducted in a prone lying position on a flat treatment table, with participants instructed to maintain a strict isometric scapular retraction until failure. The arms were placed in 90° shoulder abduction and 90° elbow flexion, resting on the table with palms facing downward. To ensure standardized execution, participants received a brief demonstration and one supervised practice attempt before formal testing. At the start of the test, participants were asked to retract their scapulae by pulling the shoulder blades together and slightly downward without elevating the shoulders or arching the thoracic spine. Once the correct position was confirmed, they were instructed to lift both forearms off the table while maintaining scapular retraction and engaging the posterior shoulder girdle, without altering trunk alignment. A calibrated digital stopwatch was used to record the total hold duration in seconds, beginning at the moment full retraction was achieved and ending when any of the following termination criteria occurred: loss of scapular retraction, visible thoracic extension or compensation, elevation of the shoulders, or voluntary cessation due to fatigue. To enhance measurement consistency, a trained physiotherapist monitored the scapular positioning throughout the test from the head end of the table. The test was performed once per participant, following a brief familiarization trial, *to* avoid muscle pre-fatigue. The SRET was selected as a pragmatic clinical measure of scapular muscle endurance because it is simple to administer and requires minimal equipment. However, evidence supporting its measurement properties in postoperative rotator cuff populations remains limited [23]. All testing procedures adhered strictly to the predefined protocol, ensuring consistency in participant positioning, test execution, and interpretation of failure criteria.

### Scapular dyskinesis

Scapular dyskinesis was assessed using a standardized visual observational method, the **Scapular Dyskinesis Test** [9], adapted from protocols described by Kibler et al.[24] and later refined by McClure et al. [25]. This test is designed to identify abnormal scapular motion during dynamic shoulder elevation tasks. It is commonly used in clinical and post-surgical rehabilitation settings due to its simplicity and high face validity [26]. Participants were instructed to hold a **1.5 kg dumbbell in each hand** (or a lighter weight if symptomatic) and perform **five consecutive repetitions of active shoulder flexion and abduction** in the scapular plane, raising the arms from neutral to full elevation and then returning to the starting position in a slow, controlled manner. Movements were performed bilaterally to allow for symmetrical visual comparison. A standardized metronome, set at 60 beats per minute (bpm), was used to ensure consistent pacing across participants. During each repetition, two trained physical therapists, blinded to patient-reported outcomes, independently observed the scapulae from posterior and lateral angles. Observers evaluated explicitly for signs of **scapular winging, dysrhythmia, early elevation, or asymmetrical movement patterns**. Classification was binary: scapular motion was rated as either **“normal” or “dyskinesis present.”** Any visible abnormality in either plane of motion was sufficient to classify the participant as having dyskinesis [26].

### Closed-chain upper extremity performance

Upper extremity functional performance was evaluated using the CKCUEST [11], a validated test for assessing shoulder girdle stability, scapular control, and neuromuscular coordination under dynamic, weight-bearing conditions. Participants assumed a standard push-up position on a non-slip mat with their hands placed 36 inches apart, marked by two parallel lines [11]. Female participants were permitted to use a modified (kneeling) push-up position if required due to post-surgical limitations. Once positioned, they were instructed to alternately reach across the midline to touch the opposite hand marker with each hand, completing as many touches as possible in 15 seconds. A full touch was defined as the hand making clear contact with the line across the body. Verbal instructions were standardized, and no feedback was given during the trial. Participants were given a single practice attempt followed by one recorded trial. The total number of touches was recorded as the CKCUEST score.

### Covariates and confounding variables

Demographic and clinical variables known to influence postoperative outcomes were recorded as covariates. These included age (years), sex (male/female), body mass index (BMI) in kg/m^2^, dominant arm involvement (yes/no), tear size (categorized as small, medium, or large based on surgical reports), and time since surgery (in months). Tear size classification was extracted from operative documentation and adhered to standard arthroscopic grading criteria. Tear size was classified intraoperatively as small (<1 cm), medium (1–3 cm), or large (3–5 cm) based on the DeOrio and Cofield criteria. Dominant arm involvement was recorded but excluded from the final regression models due to a non-significant correlation with outcomes in preliminary analyses. All covariates were assessed at baseline and adjusted for in regression and mediation models to control for potential confounding effects. The number of tendons involved, preoperative fatty infiltration (Goutallier classification), and repair configuration (single-row versus double-row) were not consistently documented in the available retrospective surgical records and therefore could not be included as covariates. As these surgical characteristics are recognised determinants of postoperative function and tendon healing, their absence should be considered when interpreting the present findings.

### Data analysis

All statistical analyses were performed using IBM SPSS Statistics for Windows, Version 24.0 (IBM Corp., Armonk, NY, USA). Before analysis, all continuous variables were examined for normality using the Shapiro–Wilk test and visual inspection of histograms and Q–Q plots, confirming that the data were normally distributed. Consequently, parametric statistical tests were applied throughout. Descriptive statistics were computed for all demographic, clinical, and outcome variables, including means and standard deviations for continuous variables, and frequencies and percentages for categorical variables. Independent samples t-tests were used to compare functional outcomes and performance-based measures between participants classified as having normal versus abnormal scapular kinematics. For categorical comparisons, including sex distribution and tear size classification, chi-square tests were conducted. To evaluate the relationships between scapular endurance, kinematic measures, and patient-reported outcomes, Pearson correlation coefficients were calculated. For predictive modeling, multiple linear regression analyses were conducted to assess the independent contributions of scapular retraction endurance, CKCUEST performance, scapular dyskinesis status, and clinical covariates (age, sex, BMI, and tear size) to ASES and DASH scores. To explore mediation effects, parametric bootstrapping procedures were applied using the PROCESS macro (Model 4) to test the indirect influence of kinematic variables on the relationship between endurance and shoulder outcomes. Scapular dyskinesis was analysed as a dichotomous mediator (present/absent). Accordingly, the mediator model (X → M) was estimated using logistic regression, whereas the outcome models (M → Y) for the continuous ASES and DASH scores were estimated using linear regression. Indirect effects were estimated using bias-corrected bootstrap confidence intervals, consistent with mediation procedures for binary mediators and continuous outcomes. Statistical significance was set at p < 0.05, and all analyses were two-tailed. Bootstrapping was performed with 5,000 resamples to derive bias-corrected 95% confidence intervals for indirect effects.

## Results

Participants had a mean age of 58.40 ± 8.90 years, with a slight female majority (53.64%) and a mean BMI of 27.30 ± 3.95 kg/m^2^ (Table 1). The dominant arm was involved in over half the cases (56.36%), and medium-sized rotator cuff tears were the most common (44.55%). The average time since surgery was 11.40 ± 3.20 months. While demographic and surgical characteristics showed no significant differences, clinically meaningful variation was evident in patient-reported outcomes: individuals reported moderate pain intensity (3.80 ± 2.10, *p* = 0.041), reduced shoulder function on the ASES scale (75.20 ± 13.50, *p* < 0.001), and functional disability on the DASH measure (28.40 ± 11.80, *p* = 0.005).

**Table 1:** Demographic and clinical characteristics of participants.

Variable	Total Sample (N = 110)	p-value
Age (years)	58.40 ± 8.90	0.451
Sex (female)	59 (53.64%)	—
BMI (kg/m²)	27.30 ± 3.95	0.289
Dominant Arm Involved	62 (56.36%)	0.127
Tear Size (Small)	38 (34.55%)	—
Tear Size (Medium)	49 (44.55%)	—
Tear Size (Large)	23 (20.91%)	—
Time Since Surgery (months)	11.40 ± 3.20	0.332
Pain Intensity (0–10 NRS)	3.80 ± 2.10	0.041
ASES Score (0–100)	75.20 ± 13.50	<0.001
DASH Score (0–100)	28.40 ± 11.80	0.005

ASES; American Shoulder and Elbow Surgeons score; BMI; Body Mass Index; DASH; Disabilities of the Arm, Shoulder and Hand; NRS; Numeric Rating Scale.

Patients classified with scapular dyskinesis indicated significantly poorer performance across all clinical and functional outcomes compared to those without dyskinesis (Table 2). Lower scores were evident in scapular muscle endurance and upper extremity stability, with dyskinesis-positive individuals showing reduced Scapular Retraction Endurance Test (mean difference: 5.40 seconds, *p* < 0.001, η^2^ = 0.130) and CKCUEST performance (mean difference: 3.20 touches, *p* < 0.001, η^2^ = 0.191). Clinically meaningful reductions in self-reported function parallelled these deficits (ASES: mean difference = 13.80, *p* < 0.001, η^2^ = 0.282) and increased disability and pain (DASH: mean difference = −15.40, *p* < 0.001, η^2^ = 0.386; NRS: mean difference = −2.00, *p* < 0.001, η^2^ = 0.220), indicating a strong association between dyskinesis and persistent postoperative shoulder impairment.

**Table 2:** Scapular endurance, kinematic measures, and patient-reported outcomes by scapular dyskinesis classification.

Variable	Dyskinesis Negative (n = 64)	Dyskinesis Positive (n = 46)	95% CI (Mean Diff)	F-value	p-value	Effect Size (η²)
Scapular Retraction Endurance Test (sec)	36.50 ± 6.80	31.10 ± 7.20	2.73 to 8.07	16.07	<0.001	0.130
CKCUEST (touches in 15s)	22.80 ± 3.40	19.60 ± 3.10	1.98 to 4.42	25.50	<0.001	0.191
ASES Score (0–100)	81.60 ± 9.80	67.80 ± 12.40	9.49 to 18.11	42.44	<0.001	0.282
DASH Score (0–100)	21.70 ± 9.20	37.10 ± 10.30	−19.13 to −11.67	67.83	<0.001	0.386
Pain Intensity (0–10 NRS)	2.90 ± 1.60	4.90 ± 2.20	−2.75 to −1.25	30.50	<0.001	0.220

ASES; American Shoulder and Elbow Surgeons score; CKCUEST; Closed Kinetic Chain Upper Extremity Stability Test; DASH; Disabilities of the Arm, Shoulder and Hand; NRS; Numeric Rating Scale; CI; Confidence Interval; η²; partial eta squared.

**Fig 1:**
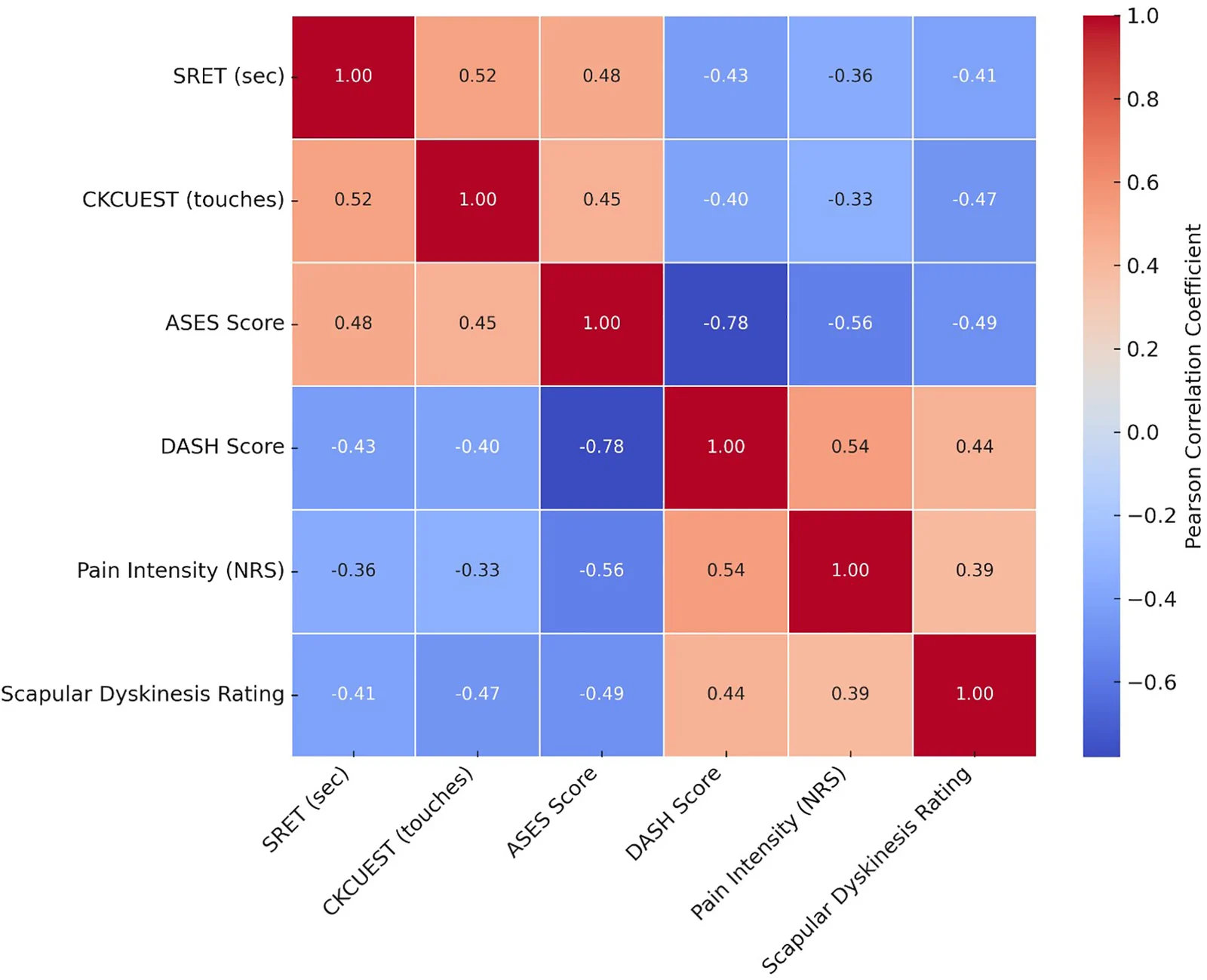
Heatmap of Pearson correlation coefficients between scapular endurance, kinematic measures, and patient-reported outcomes.

Scapular muscle endurance and upper extremity stability were moderately correlated with both improved shoulder function and reduced disability, highlighting their clinical relevance in postoperative assessment (Fig 1). Specifically, higher SRET and CKCUEST scores were positively associated with ASES scores (*r* = 0.48 and 0.45, respectively; both *p* < 0.001) and inversely correlated with DASH scores, pain intensity, and dyskinesis severity. Notably, the ASES and DASH scores showed a strong inverse correlation (*r* = −0.78, *p* < 0.001), indicating their consistent reflection of functional outcomes. Greater dyskinesis severity was associated with lower endurance, poorer upper extremity stability, reduced function, and increased pain, reinforcing its role as a clinically meaningful biomechanical impairment.

Scapular muscle endurance, upper extremity stability, and scapular kinematics were significant predictors of both shoulder function and disability, as shown in the multivariable regression model (Table 3). Greater performance on the Scapular Retraction Endurance Test and CKCUEST was independently associated with higher ASES scores (*B* = 0.42 and 0.36, respectively; both *p* ≤ 0.004) and lower DASH scores (*B* = −0.39 and −0.34; both *p* ≤ 0.011), indicating better perceived function and reduced disability. Presence of scapular dyskinesis significantly predicted lower functional scores (*B* = −4.80 for ASES) and higher disability (*B* = 5.90 for DASH), while larger tear size was also associated with worse outcomes in both models. Age had a modest negative effect on ASES (*B* = −0.18, *p* = 0.029), whereas sex and BMI were not statistically significant predictors in either model.

**Table 3:** Multivariable linear regression predicting ASES and DASH scores from scapular endurance and kinematic indicators.

Outcome	Predictor	B	SE	95% CI Lower	95% CI Upper	p-value
ASES Score	Scapular Retraction Endurance (sec)	0.42	0.09	0.35	0.61	<0.001
ASES Score	CKCUEST (touches)	0.36	0.12	0.12	0.6	0.004
ASES Score	Scapular Dyskinesis (1 = Yes)	−4.8	1.9	−8.55	−1.05	0.013
ASES Score	Age (years)	−0.18	0.08	−0.34	−0.02	0.029
ASES Score	Sex (1 = Female)	−3.1	1.7	−6.46	0.26	0.070
ASES Score	BMI (kg/m²)	−0.22	0.14	−0.49	0.05	0.106
ASES Score	Tear Size (1 = Large)	−6.2	2.3	−10.76	−1.64	0.008
DASH Score	Scapular Retraction Endurance (sec)	−0.39	0.11	−0.61	−0.17	<0.001
DASH Score	CKCUEST (touches)	−0.34	0.13	−0.6	−0.08	0.011
DASH Score	Scapular Dyskinesis (1 = Yes)	5.9	2.1	1.74	10.06	0.006
DASH Score	Age (years)	0.14	0.09	−0.04	0.32	0.126
DASH Score	Sex (1 = Female)	2.7	1.9	−1.03	6.43	0.154
DASH Score	BMI (kg/m²)	0.19	0.16	−0.13	0.51	0.238
DASH Score	Tear Size (1 = Large)	7.1	2.6	1.98	12.22	0.007

ASES; American Shoulder and Elbow Surgeons score; BMI; Body Mass Index; DASH; Disabilities of the Arm, Shoulder and Hand; CKCUEST; Closed Kinetic Chain Upper Extremity Stability Test; CI; Confidence Interval; SE; Standard Error; B; Unstandardized Beta Coefficient.

**Fig 2:**
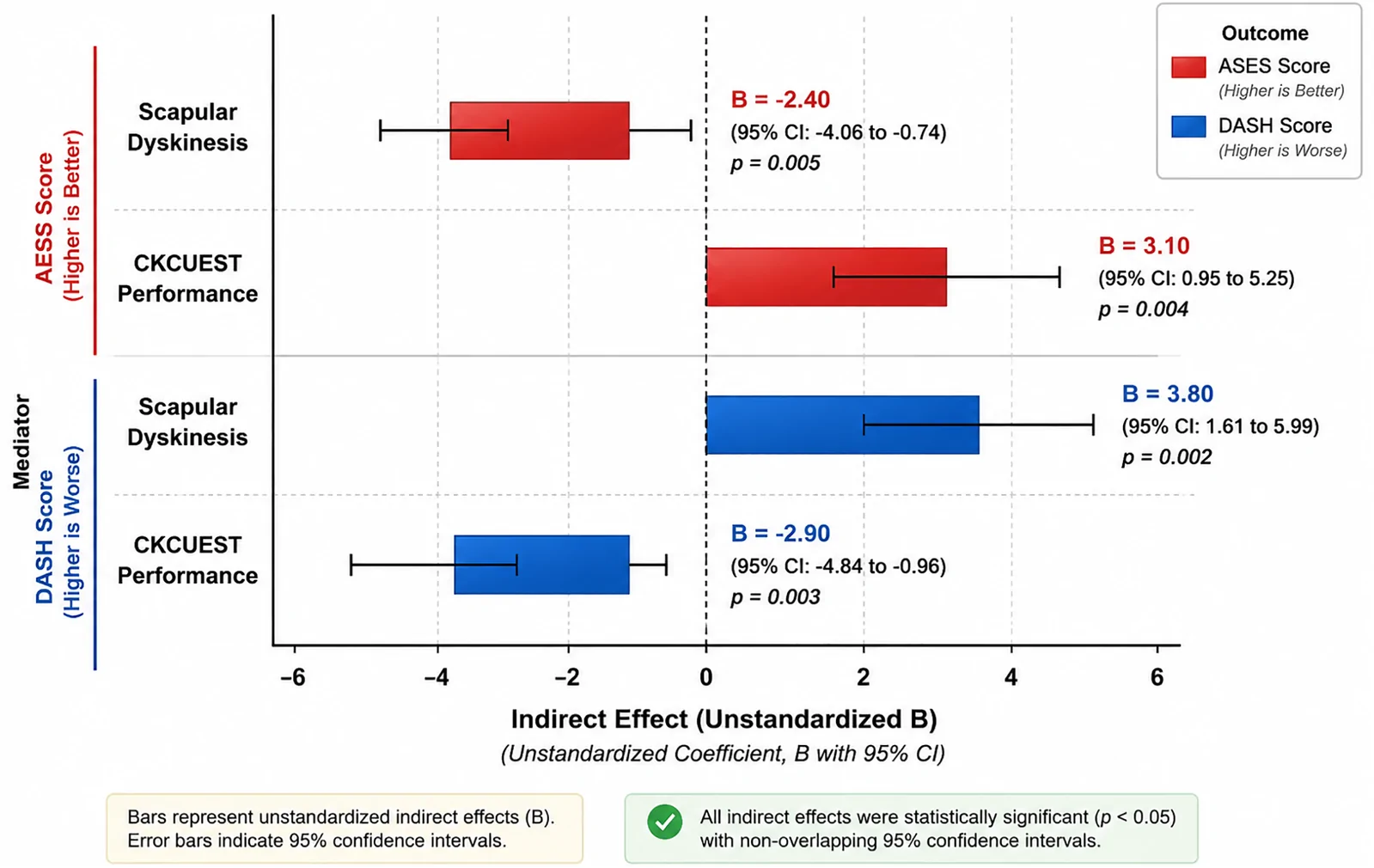
Mediation analysis showing the unstandardized indirect effects (B, 95% CI) of scapular dyskinesis and CKCUEST performance on the association between scapular endurance and ASES and DASH scores.

Both scapular dyskinesis and CKCUEST performance significantly mediated the relationship between scapular endurance and functional outcomes, indicating distinct indirect pathways influencing recovery (Fig 2). For ASES scores, the indirect effect through scapular dyskinesis was negative (*B* = −2.40, 95% CI: −4.06 to −0.74, *p* = 0.005), while mediation through CKCUEST showed a positive and statistically significant effect (*B* = 3.10, 95% CI: 0.95 to 5.25, *p* = 0.004). Similarly, in the DASH model, scapular dyskinesis mediated a significant increase in disability (*B* = 3.80, *p* = 0.002), whereas CKCUEST performance reduced perceived disability (*B* = −2.90, *p* = 0.003). All indirect effects were supported by significant Sobel tests and non-overlapping confidence intervals, highlighting the mechanistic relevance of scapular control and upper extremity stability in functional outcomes following rotator cuff repair.

**Fig 3:**
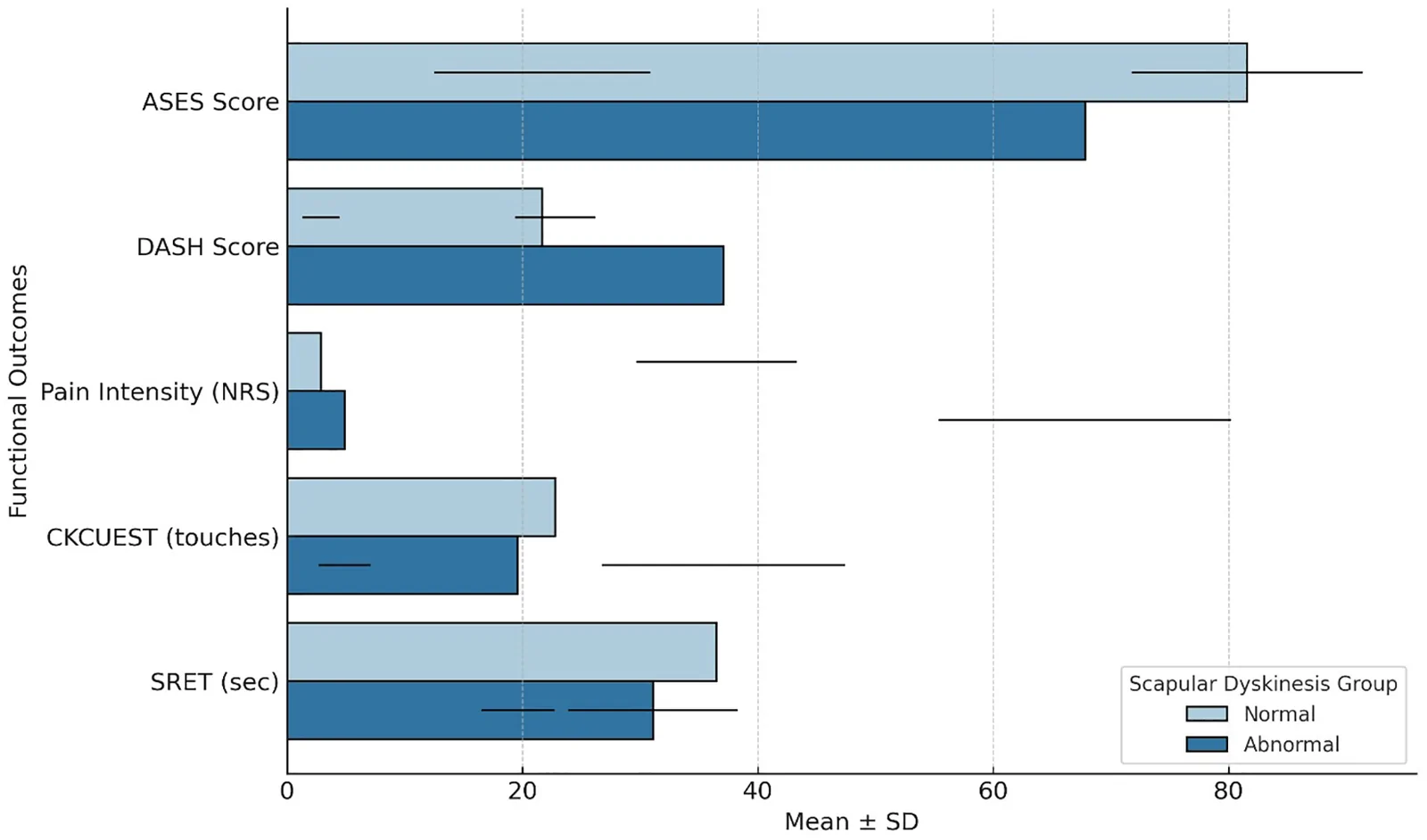
Subgroup comparison of functional outcomes between patients with and without scapular dyskinesis.

Participants with abnormal scapular dyskinesis indicated significantly poorer clinical and functional outcomes compared to those with normal scapular motion (Fig 3). Statistically significant group differences were found across all variables, with the dyskinesis group showing lower ASES scores and CKCUEST performance, as well as reduced scapular endurance (SRET), alongside higher DASH scores and greater pain intensity (all *p* < 0.001). These differences reflect a consistent pattern of greater functional impairment and symptom burden in patients with observable scapular movement abnormalities.

The post hoc power analysis confirmed that the main regression model predicting ASES and DASH scores was statistically well-powered, with an estimated power of 0.92 based on a moderate effect size (f^2^ = 0.15), α = 0.05, and a sample size of 110 (Table 4). The analysis, conducted using a fixed model with R^2^ deviation from zero and seven predictors, yielded a noncentrality parameter (λ) of 16.50, a critical F-value of 2.10, and degrees of freedom of 7 and 102 for the numerator and denominator, respectively.

**Table 4:** Post Hoc power analysis for the main regression model predicting functional outcomes.

Parameter	Value
Model	Multiple Linear Regression
Dependent Variable	ASES Score / DASH Score
Statistical Test	Fixed Model, R² deviation from zero
Effect Size (f²)	0.15
α Error Probability	0.05
Power (1 – β)	0.92
Sample Size	110
Number of Predictors	7
Noncentrality Parameter (λ)	16.50
Critical F	2.10
Numerator df	7
Denominator df	102

ASES; American Shoulder and Elbow Surgeons score; DASH; Disabilities of the Arm, Shoulder and Hand; df; degrees of freedom; f²; effect size; α; alpha level; β; beta error rate.

## Discussion

This study examined the associations of scapular muscle endurance, scapular dyskinesis, and CKCUEST performance with patient-reported shoulder function following arthroscopic rotator cuff repair. The findings indicated that reduced scapular endurance and upper extremity stability were significantly associated with greater functional disability and shoulder function. Scapular dyskinesis emerged as a key differentiator, with individuals exhibiting abnormal scapular motion showing markedly poorer performance and self-reported outcomes. Multivariable regression analysis confirmed the independent predictive value of scapular endurance, kinematic performance, and tear characteristics on both functional and disability outcomes. Mediation analysis identified indirect effects that were statistically consistent with partial mediation by scapular dyskinesis and upper extremity stability in the association between scapular endurance and postoperative outcomes. Because these analyses were based on cross-sectional data, the findings should be interpreted as exploratory statistical associations rather than evidence of causal or mechanistic pathways.

The strong association identified between scapular muscle endurance and patient-reported shoulder function supports the growing body of evidence that neuromuscular endurance is a key determinant of postoperative recovery following rotator cuff repair [4]. Scapular muscle endurance has been associated with sustained scapulothoracic control, optimal glenohumeral rhythm, and efficient force transmission during daily and overhead activities [4]. This dysfunction can limit shoulder elevation, reduce load tolerance, and exacerbate perceived disability [27]. Muench et al. [28] have emphasized that impaired scapular control has important clinical implications in individuals recovering from rotator cuff pathology. The current findings are further supported by Ciccotti et al. [29], who demonstrated that scapular muscle fatigue significantly alters upper-limb kinematics, reinforcing the importance of muscular endurance for optimal shoulder function. The consistent relationship between reduced scapular endurance and lower scores on validated functional outcome measures (ASES and DASH) underscores its clinical relevance and the need for targeted endurance training in postoperative protocols [30,31].

The mediation analysis provided additional insight into the statistical relationships among these variables, revealing that both scapular dyskinesis and CKCUEST performance partially mediated the effects of scapular endurance on shoulder function and disability [10]. These findings suggest that scapular endurance is associated with shoulder function alongside scapular motion quality and closed-chain neuromuscular control. Because all variables were measured at a single time point, the temporal sequence between scapular endurance, scapular kinematics, and functional outcomes cannot be determined, and reverse causation remains a plausible explanationl [10]. Dyskinesis reflects a disruption in scapulothoracic mechanics, which may result from or contribute to muscular fatigue and poor endurance [8]. These findings should be interpreted cautiously because the cross-sectional design precludes establishing temporal precedence. Consequently, the observed indirect effects may also reflect reverse causation or residual confounding, and confirmation of these relationships requires prospective longitudinal studies. Previous work by Song et al. [32] and Longo et al. [9] has demonstrated that altered scapular kinematics compromise shoulder performance and increase the risk of persistent dysfunction. The CKCUEST, a performance-based test of upper limb stability and control, captured deficits that align with real-world functional challenges faced by individuals with poor endurance and abnormal scapular motion [33]. The indirect effects identified in this study underscore the importance of addressing scapular mechanics and closed-chain control as integral components of comprehensive rehabilitation strategies [34]. These findings highlight the multifactorial nature of shoulder recovery, where scapular endurance interacts with motor control deficits to influence both objective performance and patient-reported outcomes [35,36].

### Clinical significance

The results of this study indicate that scapular muscle endurance, scapular kinematic function, and CKCUEST performance were associated with patient-reported shoulder function and disability following arthroscopic rotator cuff repair. The Scapular Retraction Endurance Test and CKCUEST may provide practical clinical assessments of physical performance; however, the present study did not evaluate their criterion validity, diagnostic accuracy, responsiveness, or minimal detectable change. Accordingly, these findings should be interpreted as demonstrating associations with patient-reported outcomes rather than establishing these measures as validated screening or monitoring tools.

### Limitations and areas for future research

This study has several limitations. First, its cross-sectional design precludes causal inference because exposure, mediators, and outcomes were measured simultaneously. Consequently, temporal precedence cannot be established, reverse causation cannot be excluded, and the mediation analyses performed using PROCESS Model 4 should be interpreted as exploratory statistical models rather than evidence of causal mediation. For example, poorer shoulder function may reduce participation in rehabilitation activities and thereby contribute to lower scapular endurance rather than the converse. Second, although the sample was adequately powered, it was derived from a single clinical population, which may limit the generalizability of the findings to other postoperative cohorts. Third, scapular dyskinesis was assessed using observational clinical methods that, although reliable, may not detect subtle movement abnormalities identifiable with three-dimensional motion analysis. Similarly, although the Scapular Retraction Endurance Test is readily applicable in clinical practice, evidence supporting its reliability and other measurement properties specifically in postoperative rotator cuff populations remains limited. Furthermore, neither the Scapular Retraction Endurance Test nor the CKCUEST was compared with reference standards such as instrumented strength or endurance testing or three-dimensional motion analysis, and responsiveness, minimal detectable change, and diagnostic accuracy were not evaluated. Therefore, the observed associations with patient-reported outcomes should not be interpreted as validation of these assessments for screening or longitudinal monitoring. Finally, detailed operative variables, including the number of tendons involved, Goutallier fatty infiltration grade, and repair technique (single-row versus double-row), were not consistently available from the medical records and therefore could not be included in the analyses. Residual confounding related to these surgical factors cannot be excluded. Future prospective longitudinal and interventional studies incorporating comprehensive surgical data and instrumented biomechanical assessments are warranted to establish temporal relationships among scapular endurance, scapular dyskinesis, upper extremity performance, and clinical outcomes, and to determine whether the statistically observed indirect effects represent true causal pathways.

## Conclusion

Scapular endurance and upper extremity kinematic performance were significantly associated with shoulder function and disability in individuals 6–18 months after arthroscopic rotator cuff repair. Scapular dyskinesis and CKCUEST performance partially mediated these associations, suggesting that scapular muscle performance and movement quality were statistically associated with postoperative functional outcomes. These findings support further investigation of scapular-specific clinical assessments within postoperative rehabilitation but do not establish the Scapular Retraction Endurance Test or CKCUEST as validated screening or outcome-monitoring tools.

## Supporting information

S1 File Raw data underlying the study analyses. Excel workbook containing the participant-level data used for the statistical analyses reported in this study.
(XLSX)
